# Alteration of Sphingolipids in Biofluids: Implications for Neurodegenerative Diseases

**DOI:** 10.3390/ijms20143564

**Published:** 2019-07-21

**Authors:** Luciana M. Pujol-Lereis

**Affiliations:** Centro de Investigación y Desarrollo en Inmunología y Enfermedades Infecciosas (CIDIE-CONICET), X5016DHK Córdoba, Argentina; luciana.pujol.lereis@gmail.com

**Keywords:** neurodegeneration, lipidomics, ceramides, sphingomyelin, biofluids, apoptosis

## Abstract

Sphingolipids (SL) modulate several cellular processes including cell death, proliferation and autophagy. The conversion of sphingomyelin (SM) to ceramide and the balance between ceramide and sphingosine-1-phosphate (S1P), also known as the SL rheostat, have been associated with oxidative stress and neurodegeneration. Research in the last decade has focused on the possibility of targeting the SL metabolism as a therapeutic option; and SL levels in biofluids, including serum, plasma, and cerebrospinal fluid (CSF), have been measured in several neurodegenerative diseases with the aim of finding a diagnostic or prognostic marker. Previous reviews focused on results from diseases such as Alzheimer’s Disease (AD), evaluated total SL or species levels in human biofluids, post-mortem tissues and/or animal models. However, a comprehensive review of SL alterations comparing results from several neurodegenerative diseases is lacking. The present work compiles data from circulating sphingolipidomic studies and attempts to elucidate a possible connection between certain SL species and neurodegeneration processes. Furthermore, the effects of ceramide species according to their acyl-chain length in cellular pathways such as apoptosis and proliferation are discussed in order to understand the impact of the level alteration in specific species. Finally, enzymatic regulations and the possible influence of insulin resistance in the level alteration of SL are evaluated.

## 1. Introduction

Cell membranes are composed of several hundreds of different lipid species, having lipids and proteins more or less restricted movements as a consequence of interactions between them and cytoskeletal molecules [1]. Sphingolipids (SL) are structural components of membrane lipid bilayers in eukaryotes and some prokaryotes. In particular, SL, together with cholesterol, participate in the formation of membrane microdomains in the outer leaflet of the plasma membrane that are involved in signal transduction and trafficking [2]. SL also play crucial roles as bioactive molecules in regulating cellular processes including cell proliferation and migration, apoptosis, autophagy, differentiation, senescence, and inflammatory responses [3,4,5]. For example, ceramide has been proposed to be a second messenger in diverse cellular signaling pathways, with levels of ceramides being transiently altered by several extracellular signals and physiological changes [6]. SL are highly enriched in nervous cells and they exert specific roles in modulating cell signaling, controlling neuronal survival, migration, and differentiation, responsiveness to trophic factors, synaptic stability and transmission, and neuron-glia interactions [7]. For this reason, the nervous system is particularly vulnerable to SL storage disorders [8]. Synaptic failure, even without major structural damage to neurons, seems to be a key factor of neuronal physiological decline in sphingolipidoses [9].

Increased oxidative stress is a central event in neuronal failure that induces the activation of the sphingomyelin (SM)-ceramide pathway, making SL key players in neurodegeneration. Moreover, the increase of ceramide formation from SM has been reported as specifically detrimental in neurons and oligodendrocytes compared to astrocytes and microglia, which contain higher levels of antioxidant defenses such as glutathione and manganese superoxide dismutase (MnSOD) [10]. The importance of SL in regulating neuronal and glial survival has led researchers to investigate possible level alterations of SL in neurodegenerative diseases. Biofluids such as plasma, serum, and to a lesser extent cerebrospinal fluid (CSF) are accessible sources of biomarkers, and therefore, they are chosen by researchers when investigating alterations of specific molecules in living patients with neurodegenerative diseases, including retina pathologies. The purpose of this review is to summarize and to compare results of lipidomics in biofluids of patients with neurodegenerative diseases, specifically those focused on SL species or those that found SL levels altered compared with control individuals. For a broader understanding, a brief explanation of selected lipid classes and enzymes involved in the SL metabolism is provided. Finally, the possible implication of the changes in SL species in processes associated with neurodegeneration, such as apoptosis, inflammation, and insulin resistance, are discussed, together with the potential utility of sphingolipidomic studies in basic and clinical research.

## 2. Biosynthesis of Sphingolipids

**Figure 1:**
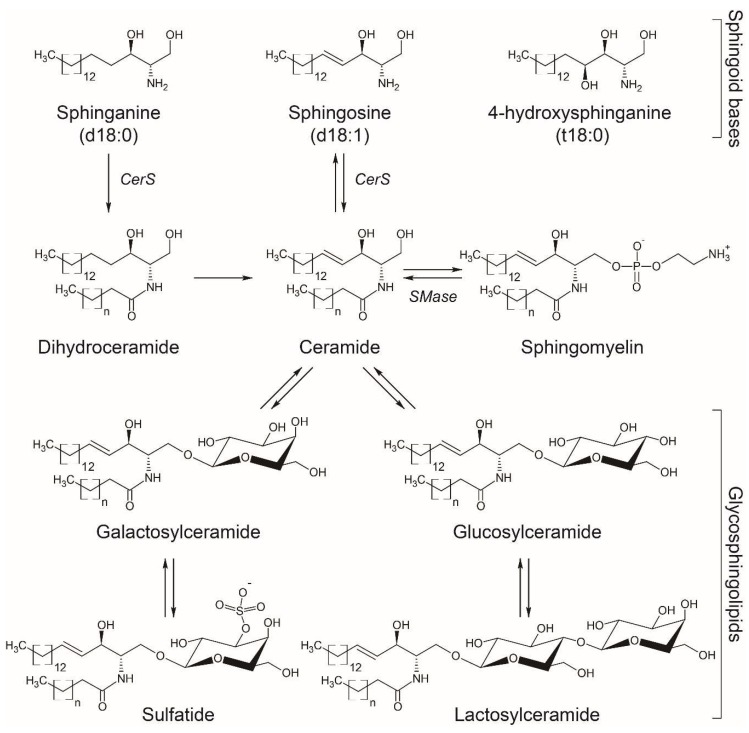
Basic structures of sphingoid bases and sphingolipids more commonly measured in biofluids. The arrows indicate the anabolic/catabolic flux, with some of the enzymes mentioned in the text indicated as follows: CerS, ceramide synthase; SMase, sphingomyelinase.

SL are characterized by the presence of a common sphingoid backbone structure. Humans have primarily sphingosine (d18:1), sphinganine or dihydrosphingosine (d18:0), and phytosphingosine or 4-hydroxysphinganine (t18:0) as sphingoid bases (Figure 1), and in small amounts the homologs d20:1, d20:0, and 6-hydroxy-sphingosine (6-t18:1), the latter one in skin [11]. The synthesis of SL involves enzymatic pathways in different cellular compartments, with ceramide playing a central role in SL metabolism [12].

The *de novo* synthesis of SL starts in the endoplasmic reticulum (ER) with the condensation of mainly serine and palmitoyl-CoA by the rate-limiting membrane enzyme serine palmitoyltransferase (SPT) to form 3-ketodihydrosphingosine, which is then reduced to generate sphinganine. Ceramide synthases (CerS) catalyze the N-acetylation of sphinganine to form dihydroceramide which is converted to ceramide by the dihydroceramide desaturase. A second pathway termed “the salvage pathway” involves the degradation of complex SL such as gangliosides in the lysosome to generate ceramides and subsequently sphingosine by ceramidases. Sphingosine can also be N-acetylated by CerS in the ER to form ceramides again (Figure 1).

**Figure 2:**
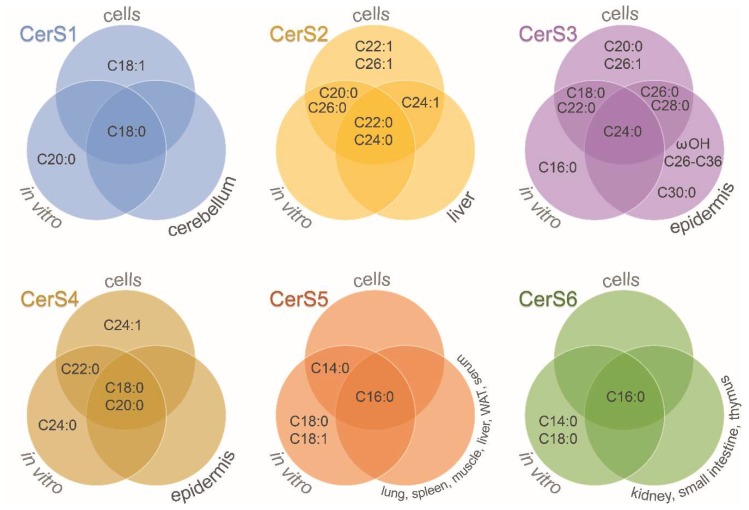
Ceramide synthases acyl-chain specificity. Venn diagrams show the results obtained for each of the six mammalian ceramide synthases (CerS) depending on the experimental approach: *in vitro* studies, overexpression or downregulation of CerS in cells, and selected tissues of CerS-deficient mice. WAT: white adipose tissue; ωOH: omega-hydroxylated acyl moieties. See more details in Table 1.

CerS are a family of enzymes comprised of six isoforms with differential specificity towards acyl-CoAs as well as tissue and cell expression [13]. Figure 2 shows the fatty acid acyl-CoA length specificity of CerS (see also Table 1). The involvement of ceramide species in selected cellular processes is discussed below (see Section 6). Additionally, each tissue has a unique expression profile of CerS. For instance, high expression of CerS1 in human brains was observed by Jiang et al. [14], and later confirmed to be the highest expressed CerS in mouse brain, followed by an intermediate expression for CerS2 and CerS4, and a lower expression for CerS5 and CerS6 [15]. Alterations in brain CerS transcript levels have been also observed during development. CerS6 expression and ceramide (Cer) C16:0 content were shown to be decreased in the adult rat brain compared to postnatal P1 brain, while other ceramide species and most CerS expression levels were increased [16]. Similarly, decreased expression of CerS2 and CerS6 was observed during mouse brain development [17]. Moreover, a specific cell type expression was shown, with neurons presenting higher CerS1 expression, while CerS2 was predominantly expressed in myelinating cells of mouse brains [17]. In mouse retina, expression of CerS1, CerS2, and CerS4 was detected, with CerS4 expressed in all retinal neurons and Müller cells [18].

A third synthesis pathway involves the hydrolysis of SM in cellular membranes to form ceramide and phosphorylcholine by sphingomyelinases (SMases). Several types of SMases have been described, with acid SMase and Mg^2+^-dependent neutral SMase being the most extensively characterized [37]. The acid SMase gene (*SMPD1*) encodes for a protein precursor that suffers differential protein modifications and trafficking, producing both the lysosomal SMase (L-SMase), and the secretory SMase (S-SMase) [38]. The degradation of SM by the acid SMase is an important source of ceramides, and L-SMase is near ubiquitously distributed in mammalian tissues, while acid SMase activity both from L-SMase and S-SMase is present in several fluids such as serum, CSF, tears, and saliva [38]. An increase in S-SMase activity in serum was observed to correlate with a parallel increase in several serum ceramide species and particularly Cer d18:1/16:0 in patients with lymphohistiocytosis [39], showing the influence of this enzyme in the content of circulating ceramides.

Ceramide can be later galactosylated in the ER to produce galactosylceramide (GalCer) or glucosylated in the cis-Golgi to produce glucosylceramide (GlcCer) [40]. These cerebrosides with distinct hexose moieties, i.e., galactose or glucose, are usually not distinguished in lipidomic studies and termed hexosylceramides (HexCer). GlcCer can be translocated to the luminal leaflet of the Golgi and trans-Golgi membranes, where addition of a galactose residue produces lactosylceramide (LacCer) [40] (Figure 1). Also in the Golgi apparatus, 3-O-sulfation of the galactose residue in GalCer produces 3-O-sulfogalactosylceramide or sulfatide, a sulfated galactocerebroside particularly abundant in the brain that participates in the formation of myelin sheath surrounding axons [41] (Figure 1). More complex SL with different glycan structures are synthetized in cells, but were not reported in the studies summarized above and exceed the scope of this review. The most common SL detected in human biofluids are ceramides, HexCer, SM, and ganglioside GM3 [42,43], though the levels of other SL such as sphinganine, sphingosine, sphingosine-1-phosphate (S1P), and sulfatide have been reported [44,45].

A review on sphingolipids faces the problem of species notation depending on the knowledge of the sphingoid base. The notation Cer C16:0 is sometimes used to include ceramides with different sphingoid bases and a fatty acyl chain of 16 carbon atoms and no double bonds. In other cases, Cer C16:0 is used exclusively for a ceramide with a sphingosine such as Cer d18:1/16:0, and dhCer C16:0 for a dihydroceramide with a sphinganine such as Cer d18:0/16:0. The method used for ceramide detection has been checked for each publication cited in this review, and when possible to clearly establish the sphingoid base, then a fatty acyl level notation (e.g., Cer d18:1/16:0) was used [46]. If it was not possible, the simpler notation Cer C16:0 was kept. In certain cases, and for comparative purposes, if it is not specified that the lipid measured or named is a dihydroceramide, the assumption was made that Cer C16:0 is a synonym for Cer d18:1/16:0. This is properly clarified throughout the review. Concerning SM species, when the sphingoid base is not known and the assumption that it has two hydroxyl groups is made, then the lipid species level notation is used, e.g., SM 34:1, that may be SM d18:1/16:0 and/or SM d18:0/16:1 [46].

## 3. Sphingolipids in Neurodegenerative Diseases: Case-Control Studies

Lipidomic studies in biofluids from patients with neurodegenerative diseases can be separated in studies with a case-control design, and longitudinal studies that focus on finding specific lipid species as predictors of a phenotypic characteristic or disease state (see Section 4).

Case-control studies have been carried out in several neurodegenerative diseases to find differences in SL species, mostly analyzing plasma samples, and in some studies measuring lipids in serum or CSF samples. In order to have a wider overview of possible changes in SL species in neurodegeneration, these studies are compared even though lipids were obtained from different fluids. From the two major classes of SL, some of the publications measured both ceramides and sphingomyelins, while others focused on one lipid class. Lipidomic or sphingolipidomic studies that reported measurement of several ceramide or SM species were included. Metabolomic studies that reported changes in one or two SL are mentioned when appropriate, but not necessarily considered for the main comparison analyses.

**Figure 3:**
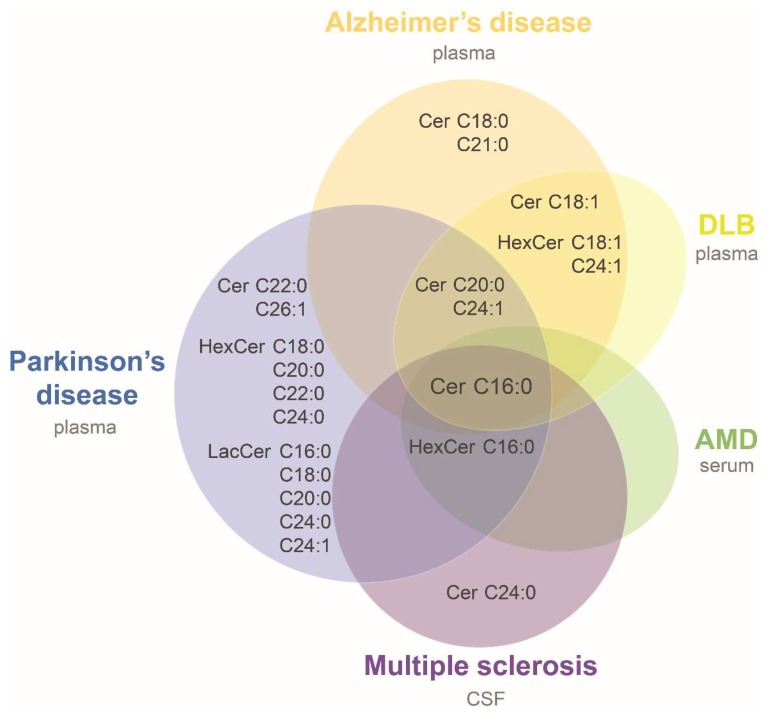
Ceramides increased in biofluids of neurodegenerative diseases. Venn diagram showing ceramide species found elevated in biofluids of patients suffering from neurodegenerative diseases compared to controls. Overlapping areas indicate species commonly altered among groups. Notation of ceramide species was simplified for the diagram (e.g., Cer C16:0 represents d18:1/16:0 ceramide). AMD: age-related macular degeneration; CSF: cerebrospinal fluid; DLB: dementia with Lewy bodies. See more details in Table 2.

Several of these studies reported level alterations on ceramide species with disease, allowing us to try a first comparison of neurodegenerative associated modifications. For this purpose, data were compiled from three studies in Alzheimer’s disease (AD) [45,47,48], and single studies in sporadic Parkinson’s disease (PD) [49], multiple sclerosis (MS) [50], dementia with Lewy bodies (DLB) [45], and age-related macular degeneration (AMD) [51]. It was assumed that ceramides and HexCer with sphingosine (d18:1) base chain were reported in those studies in which it was not clarified (i.e., [45,49]). Table 2 summarizes the main characteristics of the studies and their results. A common alteration in all seven studies was an increase in Cer d18:1/16:0 in patients compared to controls (Figure 3 and Table 2). Only two species measured in more than one study were consistently found elevated in patients: HexCer d18:1/16:0 in MS, PD and AMD, and HexCer d18:1/18:1 in the same study that evaluated AD and DLB patients compared to controls (Table 2). Cer d18:1/20:0 and d18:1/24:1 were found elevated in AD patients in one or two of the three AD studies, respectively, and in DLB and PD patients. All the other species were evaluated only in one study or were not consistently found altered in different cohorts (Figure 3 and Table 2). The higher levels of ceramide species in plasma of AD patients is in accordance with a previous study that observed higher content of total ceramides in CSF of AD patients compared with the control group [52]. Likewise, a recent work that evaluated serum SL by high-performance thin layer chromatography (HPTLC)-densitometry observed higher long and short chain ceramides in AD compared to controls [53]. The consistency of results observed in plasma, serum and CSF of AD patients supports the idea of a general biofluid increase in ceramide levels at least for this disease. Therefore, it would be important to carry out more lipidomic studies and corroborate the increase in ceramides observed in other neurodegenerative diseases.

While ceramides were consistently found increased in biofluids of patients, SM followed different patterns, although the majority of studies showed decreased levels in neurodegenerative diseases. Moreover, depending on the technique used to separate the molecules it is possible to identify the sphingoid base of each SM species, and therefore it is difficult to compare results from different studies at species level [46]. Table 3 shows the results obtained in two studies with AD patients [47,53], and single studies of PD [54], MS [55], and amyotrophic lateral sclerosis (ALS) [56]. SM species were found decreased in AD, PD and MS compared to controls (Table 3). In mammals, the major sphingoid base is sphingosine (d18:1) [57]. If we assume sphingosine (d18:1) as the major sphingoid base for the studies in which sphingoid backbone was not identified, there were lower levels of SM d18:1/24:1 in patients compared to controls in the two AD and the MS studies (Table 3).

In contrast with the results in other neurodegenerative diseases, ALS patients had elevated plasma levels of stearoyl SM (d18:1/18:0) compared to healthy controls in a metabolomics study of two independent cohorts of subjects [56]. This SL is a component of myelin sheath and may be reflecting its breakdown as part of motor neurons death [58]. Increased plasma levels of short chain SM were reported in AD compared to control subjects when evaluated by HPTLC, a result that was not corroborated in CSF using MALDI/MS-MS in the same study [53]. The comparison of results from different body fluids and the use of two different analytical techniques make it difficult to identify the reason of the discrepancy. Our lipidomics study using serum samples of AMD and control subjects observed also an increase in SM 42:2 and 42:3, possibly SM d18:1/24:1 and 24:2, specifically in geographic atrophy (GA) patients compared to controls although correction for multiple comparisons made the differences not significant [51]. This kind of correction was not done by all the studies evaluated in this review, and they would be important to be introduced in future analyses.

SL other than ceramide and SM have been found to be altered in neurological diseases. For example, Zhang et al. [59] showed higher plasma levels of ganglioside-n-acetylneuraminic acid-3 in patients with PD compared with control subjects. This lipid is a glycosphingolipid that serves as precursor to brain-abundant complex gangliosides, and higher plasma levels may be associated with reduced glucocerebrosidase activity previously reported in nervous system of PD patients [60]. Several studies reported changes in one or two SL classes in neurological diseases by techniques other than omics, but their enumeration is beyond the scope of this review.

## 4. Longitudinal Studies Evaluating SL as Predictors of Neurological Phenotypes or Disease Incidence

The Women’s Health and Aging Study II carried out in Baltimore is a population-based longitudinal study of older women followed up to nine years, and used by Mielke and coworkers [44,61] to evaluate baseline serum levels of SM and ceramide species as predictors of subsequent cognitive impairment, or to find associations with an increased risk of all-cause dementia and AD. Researchers showed that total SM and Cer d18:1/16:0, 18:0, 22:0, 24:1, 24:0, and sulfatide were suited to predict impairment on delayed memory recall, Cer d18:1/16:0, 20:0, and 22:0 predicted impairment on immediate memory recall, and higher levels of Cer d18:1/16:0, and 20:0 were associated with decreased psychomotor speed [44]. Moreover, Cer d18:1/16:0, 24:0, and LacCer were associated with a higher risk of AD [61]. In contrast, the Baltimore Longitudinal Study of Aging showed no association between ceramide levels and risk of AD in women, but demonstrated that higher levels of SM d18:1/16:0, and 22:1 at baseline reduced the risk of AD for this gender [62]. Among men, higher levels of Cer d18:1/16:0, 18:0, 22:0, and 24:0, and SM d18:1/18:0, 18:1, 20:1, and 22:1 were associated with increased risk of developing AD [62]. Interestingly, higher contents of Cer d18:1/16:0 have been consistently associated with neurodegeneration both in case-control and longitudinal studies.

A serum non-targeted metabolomics approach of samples from mild cognitive impairment (MCI) and age-matched AD subjects showed that sphinganine-1-phosphate (dihydro-S1P) can predict the conversion of MCI to probable AD [63]. Both S1P and dihydro-S1P act extracellularly via S1P receptors, although S1P but not dihydro-S1P plays an intracellular specific role as suppressor of apoptosis [64]. Further research is needed to understand the possible influence of dihydro-S1P levels in neurodegeneration.

## 5. Sphingolipids at the Site of Neurodegeneration

The changes in SL levels observed in plasma and CSF may be reflecting alterations in nervous tissues undergoing degeneration. In accordance with the results observed in plasma, AD patients presented elevated levels of ceramides, and in some cases GlcCer and GalCer, compared with control donors in white matter of temporal cortex and cerebellum [65], pre frontal cortex (PFC) [66], middle frontal gyrus, which is a brain region with extensive Aβ plaques and neurofibrillary tangles, and in isolated membranes from brain tissue samples [67]. Other studies evaluated changes in SL levels in specific disease-associated structures or cell types. Astroglia with ceramide-immunoreactivity was detected in frontal cortices of AD brains but not control brains, and this reactivity colocalized with senile plaques [52]. A later study showed a specific enrichment of senile plaques in saturated ceramides Cer d18:1/18:0 and Cer d18:1/20:0 with respect to adjacent plaque-free neuropil [68].

In the anterior cingulate cortex (ACC) of PD patients a shift from Cer d18:1 longer (C24:1, C23:0, C22:0) to shorter (C16:0, C18:0, C18:1, C20:0) acyl chain composition was observed compared to control ACC [69]. These shifts in the composition of ceramide fatty acids may be associated with regulation of CerS or SMase. CerS1 is the prevalent ceramide synthase in the brain [70], resulting in Cer d18:1/18:0 being the predominant ceramide species in the central nervous system, while CerS2 is expressed in neurons and in oligodendrocytes during the myelination process [71]. CerS2 null mice showed decreased levels of very-long-chain (VLC) Cer d18:1/24:0 and 24:1 with a compensatory increase in Cer d18:1/16:0 and 20:0 in the brain [71]. The increase in brain tissue of Cer d18:1/16:0 is in accordance with the finding that this ceramide species was found elevated in most of the case-control studies (Figure 3). Regarding SM levels in AD brains compared with controls, it was shown that there was a down-regulation of long-chain (LC) SM (d18:0/20:0 and d18:1/20:0) and upregulation of VLC SM (d18:1/22:1 and d18:1/26:1) in PFC, a reduction in LC SM levels (including d18:0/18:0, d18:1/16:1, and d18:1/18:0) in the entorhinal cortex (ERC) [66], and of SM d18:1/24:0 levels in middle frontal gyrus [67]. In general, decreased levels of biofluid SM species were reported in AD and other neurodegenerative diseases (Table 3).

Brain SL are also abundant in the monounsaturated nervonic acid (24:1n-9) in comparison with other tissues, and the proportion of this LC fatty acid was reported to be lower in white matter and myelin samples of MS donors compared with controls [72], similarly to the lower levels of SM d18:1/24:1 and other SM species observed in MS CSF samples (Table 3). This is in accordance with the fact that MS pathology involves demyelination of axons and oligodendrocyte loss [73]. No changes in SM, ceramide and HexCer species with nervonic acid were observed in the gray matter of mid-frontal cortex in the brain of subjects with AD compared with control subjects [74], although higher levels of Cer and HexCer and lower SM containing nervonic acid were detected in AD biofluids (Table 2 and Table 3). In the same manner, it was previously reviewed that levels of cerebrosides and sulfatide were found increased, decreased or unchanged in AD brains compared with controls depending on the sample being taken from cortex or hippocampus, white or gray matter [75]. This may be associated with the specific pathology affecting certain brain structures, and/or with the sample preparation [43]. Interestingly, Satoi and coworkers [52] demonstrated that retinoblastoma NB2a cells undergoing apoptosis due to treatment with retinoic acid presented higher ceramides and SM content inside the cells, and a specific extracellular release of ceramide but not SM. This differential release of SL species may influence the inconsistency observed in the tendency of SM levels in neurodegeneration. Further studies are necessary to understand these discrepancies.

## 6. Specific Regulation and Properties of Lipid Species According to Fatty Acid Chain

Most of the lipidomic studies presented in this review showed Cer d18:1/16:0 associated with disease or cognitive impairment. Lipid micelles containing Cer C16:0, and to a lesser extent Cer C24:0, were shown to induce oxidative stress and decrease neuronal maximal respiratory rate and the spare respiratory capacity in primary rat hippocampal neurons, while Cer C22:0 or no addition of ceramide had no effect [50]. Accordingly, ceramides with fatty acyl chains of 16 and 24 carbon atoms were shown to induce apoptosis of neutrophils via caspase activation [76], and Cer d18:1/16:0 has been associated with TNF-alpha-induced apoptosis in rodent hepatocytes [77]. Cer C16:0 conjugated with BSA was also found to cause cell death in a dose dependent manner in mouse retina-derived 661W cells [78]. Another study that used a novel technique termed traceless ceramide ligation demonstrated that Cer d18:1/16:0 or 18:0 delivered into HeLa cells resulted in a reduction of cell viability compared to sphingosine, while no reduction was observed with monounsaturated Cer d18:1/18:1 or 24:1 [79].

Grösch et al. [80] hypothesized that the capacity of Cer C16:0 to mix well with cholesterol and therefore influence the functionality of lipid rafts may explain the ability of ceramide-enriched platforms to activate both intrinsic and extrinsic apoptotic pathways. On the other hand, Cer C24:0 does not mix properly with cholesterol, which may explain its proliferative effects [81,82]. Following the same tendency, another study demonstrated that the BCL-2 family member BAK is required for generation of LC ceramides (C16-C18), but not VLC ceramides (C24), and the subsequent induction of apoptosis as a consequence of the formation of mitochondrial ceramide-rich domains that is involved in the BAX/BAK translocation [83].

As suggested before, regulation of SMases may cause the alteration of SL species observed in neurodegeneration. A recent study in a chronic psychosocial stress mice model, which is a risk factor for inflammatory disorders and major depressive disorder among others, showed that stressed mice presented an increase in hepatic and S-SMase activity and increased Cer d18:1/16:0 and decreased Cer d18:1/24:0 content in the liver [84]. In patients with hemophagocytic lymphohistiocytosis, a systemic inflammatory syndrome, serum S-SMase activity was elevated compared to controls, with a parallel increase of Cer d18:1/16:0, but a decrease of Cer d18:1/24:0 concentrations [39]. Interestingly, it was shown in MCF7 breast carcinoma cells that IL-1β induced up-regulation of S-SMase secretion and increased the selective production of Cer d18:1/16:0 and d18:0/16:0, whereas higher L-SMase activity was associated with a selective increase in VLC ceramides such as Cer d18:1/26:1, suggesting distinct metabolic roles for each acid SMase [85]. Finally, Pieragostino et al. [55] suggested that the decrease in SM species observed in MS patients compared to controls (Table 3) may be a consequence of the higher acid SMase activity and the number of acid SMase enriched exosomes in CSF of MS patients, which correlated with disease severity.

The shifts from VLC to LC ceramides in patients and mice models has been also suggested to be a consequence of a balance or compensation in CerS activities. A study using HeLa cells demonstrated that knockdown of *CERS2* produces a shift in ceramide composition from C24 to C16 species conferring greater susceptibility to apoptosis induced by cisplatin, UV, or C6 ceramide [86]. Similarly, an inter-regulation of SL species was demonstrated by knockdown of CerS in MCF-7 cells. Downregulation of CerS2 resulted in decreased levels of Cer containing C22–C24 saturated and monounsaturated fatty acids, but a large increase in SL containing C16:0, and to a lesser extent C14:0 and C18:0 fatty acyl chains. On the other hand, CerS6 knockdown decreases Cer d18:1/16:0 and d18:0/16:0 [22]. Down-regulation of CerS2 in SMS-KCNR neuroblastoma cells causes decrease in VLC Cer d18:1/24:0 and 24:1 and increase in LC Cer d18:1/14:0 and 16:0, accompanied with activation of the unfolded protein response (UPR), induction of autophagy and growth arrest, but not apoptosis [87]. The authors also showed that the increase of LC ceramides in CerS2-down-regulated cells may be caused by reverse ceramidase activity of alkaline ceramidases 1 and 2 that catalyze the synthesis instead of the hydrolysis of ceramides, but not by CerS5 and CerS6 activities [87].

However, to what extent the fatty acyl chain of ceramides is responsible of certain cellular responses is still under discussion. CerS1-deficient mice presented decreased levels of Cer d18:1 with C18 acyl chain length and increased levels of species with other acyl chains, i.e., C16, C20, C22, and C24, compared to wild-type mice, and these changes were accompanied with neuronal apoptosis in the cerebellum [26]. In older mice, the authors also detected an elevation of free sphingosine and sphinganine levels [26], and in a further study they suggested that the accumulation of these sphingoid bases rather than the reduction in C18 ceramides, may cause the observed neuronal death [88].

## 7. Ceramides and Insulin Resistance in Neurodegeneration

Ceramides have been associated with the development of insulin resistance [89]. Obese humans presented elevated *CERS6* mRNA expression and Cer C14:0, C16:0, C16:1, C18:0, C18:1, and C22:1 levels in adipose tissue compared to lean subjects, and a correlation between increasing *CERS6* expression and insulin resistance [90]. Accordingly, Cer d18:1/18:0, 20:0, 24:1, and total ceramide levels were higher in obese subjects with type 2 diabetes compared to lean healthy controls, and ceramide levels correlated with the severity of insulin resistance [91]. Cer d18:1/16:0 was not measured in the later study. A connection between circulating ceramides and skeletal muscle insulin sensitivity has been proposed in obese and type 2 diabetes patients [92,93], with LDL-ceramide as a possible linker between the liver and muscles [94], an important association that may explain elevation of biofluid SL in disease. Similar to the observations in humans, CerS6-deficient mice showed reduced Cer C16:0 content and protection from high-fat diet-induced obesity and glucose intolerance [90], while CerS5 knock-out mice under a high-fat diet exhibited a reduction of Cer d18:1/16:0 in white adipose tissue and improved glucose tolerance compared to wild type mice [35]. A recent study evaluated hepatic lipids in about 100 genetically diverse inbred strains of mice fed on a high-fat/high-sucrose diet, and found that females were less insulin resistant but had higher Cer d18:1/16:0 levels than males, and only Cer d18:1/20:0 levels were increased in males and were associated with insulin resistance specifically for this sex [95].

Insulin resistance has also been implicated in some of the neurodegenerative diseases reviewed here in which higher circulating ceramide levels were reported. The hippocampus has a high glucose demand and is an insulin-sensitive tissue, making it susceptible to insulin resistance during aging [96]. Accordingly, abnormalities in glucose regulation and insulin resistance are chronic metabolic disorders that have been recognized to contribute to late onset AD [97,98]. In this context, high glycemic index (GI) of the diet and diabetes have been implicated as risk factors for AMD [99,100]. Although the uptake of glucose in retinal pigment epithelium (RPE) cells is not insulin-dependent, the signaling pathways modulated by the insulin receptor (IR) in RPE cells regulate the generation of reactive oxygen species and the expression of pro-inflammatory cytokines in mice retina [101]. Considering that ceramide is proposed to mediate insulin-resistance in skeletal muscle by blocking Akt signaling [94], it may be possible that higher ceramide levels in AMD influence the retinal homeostasis in part through an alteration of insulin signaling in the RPE. This may also impact insulin-dependent activation of mammalian target of rapamycin (mTOR) in RPE, which is implicated in cellular metabolism, protein synthesis, and cellular growth, among other processes [102]. On the other hand, it was demonstrated that the photoreceptor, inner nuclear and ganglion cell layers of frog and rat retinas express the insulin stimulated-glucose transporter Glut4 [103], although a study of Glut transporters in the human eye failed to detect Glut4 expression [104].

## 8. Conclusions and Perspectives

The main goal of this review was to have an outlook on SL alterations in biofluids of neurodegenerative diseases. A common finding in all the studies evaluated was an increase in ceramide species in different biofluids of patients compared to controls, with Cer d18:1/16:0 as the main species altered in a recurrent manner. These results evince that individual ceramide species may be considered general markers of neurodegeneration, rather than disease specific. However, it might be useful to make use of standardized methods for the measurement of SL in biofluids so as to enable the subsequent analysis of a common panel of SL species by innovative bioinformatics tools and to find disease-specific patterns. In this regard, two recent studies applied multivariate and bioinformatic analyses to differentiate between healthy individuals and patients according to several SL species. After applying unsupervised machine-learning to analyze serum concentrations of three lipid classes from MS patients and healthy controls, Lötsch et al. [105] found that the data structures emerging from the ceramide species analysis were able to be almost completely distinguished between the two groups of individuals, with about 95% of accuracy. In another work, unsupervised methods of data structure detection demonstrated that seven plasma SL mediators were able to diagnose dementia with an accuracy of 77%, in contrast to the 65% or less accuracy obtained by using random lipid markers [106]. A later analysis by Lötsch et al. [107] combined eight lipid species, including three SL, a lysophospholipid, an eicosanoid, a pterin and two endocannabinoids, reaching a high accuracy of 96% to detect the presence or absence of MS. These kinds of methodologies may be useful to establish alteration patterns according to specific diseases, or disease types. Our study in AMD showed that Cer d18:1/16:0, and to a lesser extent SM 42:2 and 42:3, were increased specifically in GA patients, while HexCer d18:1/16:0 was increased in GA and choroidal neovascularization (CNV) patients compared to controls [51]. These differences between GA and CNV together with alterations in other SL species are likely to contribute to specific disease types patterns. Glycosphingolipids have been previously implicated in angiogenesis, the process that causes vessel proliferation in patients with CNV. Inhibition of LacCer synthase expression in human umbilical vein endothelial cells was shown to inhibit angiogenesis [108], and the use of antibodies against globotriaosylceramide inhibited tumor-induced angiogenesis in mouse neoplastic lesions [109]. It would be interesting to measure more complex glycoceramides in AMD patients to verify if LacCer or other glycosylated ceramides are specifically increased in CNV and may be regarded as possible therapeutic targets or neovascularization prognostic markers.

The results from sphingolipidomics in biofluids are also useful so as to understand mechanisms involved in the pathogenesis of diseases and their connection to other markers such as genetic and environmental factors. We found that genetic variants in *CFH* and *ARMS2* genes may influence the association between Cer d18:1/16:0 and AMD, whereas genetic variants in genes involved in lipid transport and metabolism such as *APOE*, *LIPC*, and *LPL* may not [51]. Han et al. [47] showed that AD patients carrying APOE4 allele(s) had lower plasma SM levels than those with other isoforms, although this association was not present in controls. Similar results had previously been observed in ApoE4 AD brains, in which lower SM and higher ceramide and sulfatide levels were reported in comparison with ApoE3 AD brains, a tendency that was not replicated in brain tissues from normal subjects [110]. Therefore, although validations are needed, associations observed between plasma lipids and genetic variants may have a correlation in the tissues undergoing neurodegeneration.

Technical limitations for the comparison of SL levels in biofluids have arisen from the use of different platforms that are able to measure different panels of SL classes and species. Furthermore, the understanding and comparison of SM results were complicated by the non-identification of sphingoid bases. In order to translate findings to clinical approaches, such as disease specific SL patterns, common analytical methods need to be considered in the future.

## Figures and Tables

**Table 1:** Ceramide synthases acyl-chain specificity. Ceramide synthases (CerS) acyl-CoA preferences for the synthesis of ceramides according to different experimental approaches.

	Functional Studies in Cells				*In vitro* Studies			CerS-Deficient Mice Studies		
CerS	Acyl	Species	Cell Lines	Ref.	Acyl	Species	Ref.	Acyl	Tissue	Ref.
**CerS1**	C18:0 C18:1	mouse human	HEK 293T, HeLa, UM-SCC-22A, MCF-7, K562, A549	[19,20,21,22,23,24]	C18:0 C20:0	mouse human	[21,25]	C18:0	cerebellum	[26]
**CerS2**	C20:0 C22:0 C22:1 C24:0 C24:1 C26:0 C26:1	mouse human	HEK 293T, HeLa, MCF-7, Yeast	[15,22,27,28]	C20:0 C22:0 C24:0 C26:0	mouse human	[15,25,29]	C22:0 C24:0 C24:1	liver	[30]
**CerS3**	C18:0 C20:0 C22:0 C24:0 C26:0 C26:1 C28:0	mouse human	HEK 293T, HeLa, MCF-7, Yeast	[22,28,31]	C16:0 C18:0 C22:0 C24:0	mouse	[31]	C24:0 C26:0 ωOH-C26:0 * C28:0 C30:0 ωOH-C32:0 ωOH-C32:1 ωOH-C34:0 ωOH-C34:1 ωOH-C34:2 ωOH-C36:1	epidermis	[28]
**CerS4**	C18:0 C20:0 C22:0 C24:1	mouse human	HEK 293T, MCF-7	[22,32]	C18:0 C20:0 C22:0 C24:0	mouse human	[25,29,32]	C18:0 C20:0	epidermis	[33]
**CerS5**	C14:0 C16:0	mouse human	HEK 293T, HeLa, A549	[20,24,27,32]	C14:0 C16:0 C18:0 C18:1	mouse	[25,32,34]	C16:0	lung, spleen, muscle, liver, white adipose tissue, serum	[35]
**CerS6**	C16:0	human	MCF-7, K562, A549	[22,23,24]	C14:0 C16:0 C18:0	mouse	[25]	C16:0	kidney, small intestine, thymus	[36]

* ωOH: omega-hydroxylated acyl moieties.

**Table 2:** Comparison of case-control lipidomic studies reporting alterations of ceramide species in neurodegenerative diseases.

Disease		Alzheimer’s Disease (AD)			Parkinson’s Disease (PD)	Multiple Sclerosis (MS)	Dementia with Lewy Bodies (DLB)	Age-Related Macular Degeneration
Reference		[47]	[45]	[48]	[49]	[50]	[45]	[51]
Matrix		Plasma	Plasma	Plasma	Plasma	CSF	Plasma	Serum
Cohort	State/type of disease (*n*)	Mild or moderate * (26)	High or intermediate likelihood ** (18)	Late onset (202)	PD-CN, PD-MCI, PDD (52)	CIS, RRMS, PPMS, PRMS (13)	High-likelihood ***, with LBs (13)	CNV and GA (244)
	criteria for controls (*n*)	cognitively normal (26)	cognitively normal (21)	cognitively normal (207)	cognitively normal (5)	no MS (10)	cognitively normal (21)	no other eye disease (129)
Platform		ESI/MS/MS	ESI/MS/MS	UPLC/MS	LC/ESI/MS/MS	HPLC/ESI/MS/MS	ESI/MS/MS	ESI/MS/MS
Statistics		Wilcoxon rank sum test	Kruskal-Wallis rank test	Generalized linear model	Mann-Whitney U tests	none	Kruskal-Wallis rank test	Linear regression
Covariates		no	no	yes ^#^	no	no	no	yes ^##^
Multiple comparison’s correction		no ^†^	no	no	no	no	no	yes
**Ceramides**								
Cer d18:1/16:0		↑	↑	↑	↑	(↑)	↑	↑ (GA)
Cer d18:1/18:0 ^‡^		ns	ns	↑	ns	ns	ns	ns
Cer d18:1/20:0 ^‡^		ns	↑	ns	↑	ns	↑	ns
Cer d18:1/21:0		↑						
Cer d18:1/22:0		ns		ns	↑	ns		ns
Cer d18:1/23:0		ns						ns
Cer d18:1/24:0		ns	ns	ns	ns	(↑)	ns	ns
Cer d18:1/26:0		ns			ns			
Cer d18:1/18:1		ns	↑				↑	
Cer d18:1/22:1		ns			ns	(↑)		
Cer d18:1/24:1		ns	↑	↑	↑	ns	↑	ns
Cer d18:1/26:1		ns			↑			
Cer d18:1/28:0, 23:1, 28:1, 22:2, 24:2, 28:2 ^‡^		ns						
**Monohexosylceramides (Gluco- and Galactosylceramides)**								
HexCer d18:1/16:0					↑	(↑)		↑
HexCer d18:1/18:0			ns		↑	ns	ns	
HexCer d18:1/20:0					↑	ns		
HexCer d18:1/22:0					↑	ns		
HexCer d18:1/24:0					↑	ns		
HexCer d18:1/26:0					ns			
HexCer d18:1/16:1					↑			
HexCer d18:1/18:1			↑				↑	
HexCer d18:1/22:1					ns	(↑)		
HexCer d18:1/24:1			↑		ns	ns	↑	ns
HexCer d18:1/26:1						ns		
Lactosylceramides								
LacCer 16:0, 18:0, 20:0, 24:0, 24:1					↑			

* Mini-Mental State examination (MMSE); ** National Institute of Aging (NIA)-Reagan Criteria; *** third report of the DLB consortium; ^#^ center of origin of the sample, gender, age, APOE status, batch, internal standard; ^##^ gender, age, batch. ^†^ q values (cumulative false discovery rates) re-evaluated. ^‡^ 2′-hydroxy N-acyl chain ceramides were also identified but no significant differences were detected; OH_N16:0/N18:0 and OH_N19:1/N20:0 species were not resolved. CIS: clinically isolated syndrome; CNV: choroidal neovascularization; CSF: cerebrospinal fluid; ESI: electrospray ionization; GA: geographic atrophy; HPLC: high performance liquid chromatography; LC: liquid chromatography; MS (in platform): mass spectrometry; PD-CN: cognitively non-affected PD; PDD: PD-dementia; PD-MCI: PD-mild cognitive impairment; PPMS: primary-progressive MS; PRMS: progressive-relapsing MS; RRMS: relapsing-remitting MS; UPLC: ultra-performance liquid chromatography. ↑: significantly increased in disease subjects; ns: non-significantly altered; (↑): increased in MS patients according to the authors, no statistics performed. Empty cells indicate that the lipid species was not evaluated in the corresponding study.

**Table 3:** Comparison of case-control lipidomic studies reporting alteration of sphingomyelin (SM) species in neurodegenerative diseases.

Disease		Alzheimer’s Disease (AD)		Parkinson’s Disease (PD)	Multiple Sclerosis (MS)	Amyotrophic Lateral Sclerosis (ALS)
Reference		[47]	[53]	[54]	[55]	[56]
Matrix		Plasma	CSF	Plasma	CSF	Plasma
Cohort	State/type of disease (*n*)	Mild or moderate * (26)	Mild or moderate * (16)	Early disease state (77)	CIS, RRMS, PPMS (20)	Study 1 (62) Study 2 (99)
	criteria for controls (*n*)	cognitively normal (26)	cognitively normal (10)	cognitively normal (69)	OND (17)	cognitively normal Study 1 (69) Study 2 (48)
Platform		ESI/MS/MS	MALDI/MS-MS	UHPLC/QTOF/MS	LC-ESI-MS/MS	UHPLC/MS/MS
Sphingoid base identified		no	yes	no	yes	no
Statistics		Wilcoxon rank sum test	Kruskal-Wallis; best separation peaks	PLS and RF selection followed by univariate tests	PLS-DA; unpaired *t*-test	Welch’s two-sample t-test
Covariates		no	no	age, gender	no	no
Multiple comparison’s correction		no ^†^	no	yes	no	yes
Sphingomyelins						
*Sphingoid base not identified:* *N-linked fatty acid*						
SM N17:1, 18:0, 24:2		ns				
SM N20:0, 21:0, 22:0, 23:0, 24:0, 22:1, 23:1, 24:1		↓				
*Sphingoid base not identified: Hydroxyl Group Level*						
SM d30:1, 32:1, 39:1				(↓)		
*Sphingoid base identified*						
SM d18:1/13:0, 14:0, 16:0, 16:1 (9Z)(OH)					↓	
SM d18:1/18:0						↑
SM d18:1/24:1 (15Z)			↓		↓	
SM d18:2/20:0, 22:1					↓	

* Mini-Mental State examination (MMSE). ^†^ Q values (cumulative false discovery rates) were evaluated. CIS: clinically isolated syndrome; CSF: cerebrospinal fluid; ESI: electrospray ionization; LC: liquid chromatography; MALDI: matrix-assisted laser desorption/ionization; MS (in platform): mass spectrometry; PPMS: primary-progressive MS; QTOF: quadrupole time-of-flight; RRMS: relapsing-remitting MS; UHPLC: ultra high performance liquid chromatography. Statistics. PLS-DA: partial least square-discriminant analysis; RF: random forest. ↑/↓: significantly increased/decreased in disease subjects; ns: non-significantly altered; (↓): SM species significantly altered between controls and PD patients according to the PLS model, but with p-values > 0.05 in subsequent Welch’s t-test or Wilcoxon test false discovery rate (FDR) adjusted. Empty cells indicate that the lipid species was not evaluated in the corresponding study.

## References

[1] Lingwood D., Simons K. (2010). Lipid rafts as a membrane-organizing principle. Science.

[2] Olsen A.S.B., Færgeman N.J. (2017). Sphingolipids: Membrane microdomains in brain development, function and neurological diseases. Open Biol..

[3] Hannun Y.A., Obeid L.M. (2008). Principles of bioactive lipid signalling: Lessons from sphingolipids. Nat. Rev. Mol. Cell. Biol..

[4] Van Brocklyn J.R., Williams J.B. (2012). The control of the balance between ceramide and sphingosine-1-phosphate by sphingosine kinase: Oxidative stress and the seesaw of cell survival and death. Comp. Biochem. Physiol. B Biochem. Mol. Biol..

[5] Nixon G.F. (2009). Sphingolipids in inflammation: Pathological implications and potential therapeutic targets. Br. J. Pharmacol..

[6] Venkataraman K., Futerman A.H. (2000). Ceramide as a second messenger: Sticky solutions to sticky problems. Trends Cell Biol..

[7] Piccinini M., Scandroglio F., Prioni S., Buccinna B., Loberto N., Aureli M., Chigorno V., Lupino E., DeMarco G., Lomartire A. (2010). Deregulated sphingolipid metabolism and membrane organization in neurodegenerative disorders. Mol. Neurobiol..

[8] Sural-Fehr T., Bongarzone E.R. (2016). How membrane dysfunction influences neuronal survival pathways in sphingolipid storage disorders. J. Neurosci. Res..

[9] Cantuti-Castelvetri L., Bongarzone E.R. (2016). Synaptic failure: The achilles tendon of sphingolipidoses. J. Neurosci. Res..

[10] Jana A., Hogan E.L., Pahan K. (2009). Ceramide and neurodegeneration: Susceptibility of neurons and oligodendrocytes to cell damage and death. J. Neurol. Sci..

[11] Zheng W., Kollmeyer J., Symolon H., Momin A., Munter E., Wang E., Kelly S., Allegood J.C., Liu Y., Peng Q. (2006). Ceramides and other bioactive sphingolipid backbones in health and disease: Lipidomic analysis, metabolism and roles in membrane structure, dynamics, signaling and autophagy. Biochim. Biophys. Acta Biomembr..

[12] Mullen T.D., Hannun Y.A., Obeid L.M. (2012). Ceramide synthases at the centre of sphingolipid metabolism and biology. Biochem. J..

[13] Cingolani F., Futerman A.H., Casas J. (2016). Ceramide synthases in biomedical research. Chem. Phys. Lipids.

[14] Jiang J.C., Kirchman P.A., Zagulski M., Hunt J., Jazwinski S.M. (1998). Homologs of the yeast longevity gene LAG1 in *Caenorhabditis elegans* and human. Genome Res..

[15] Laviad E.L., Albee L., Pankova-Kholmyansky I., Epstein S., Park H., Merrill A.H., Futerman A.H. (2008). Characterization of ceramide synthase 2: Tissue distribution, substrate specificity, and inhibition by sphingosine-1-phosphate. J. Biol. Chem..

[16] Novgorodov S.A., Chudakova D.A., Wheeler B.W., Bielawski J., Kindy M.S., Obeid L.M., Gudz T.I. (2011). Developmentally regulated ceramide synthase 6 increases mitochondrial Ca^2+^ loading capacity and promotes apoptosis. J. Biol. Chem..

[17] Becker I., Wang-Eckhardt L., Yaghootfam A., Gieselmann V., Eckhardt M. (2008). Differential expression of (dihydro)ceramide synthases in mouse brain: Oligodendrocyte-specific expression of CerS2/Lass2. Histochem. Cell Biol..

[18] Bruggen B., Kremser C., Bickert A., Ebel P., Vom Dorp K., Schultz K., Dormann P., Willecke K., Dedek K. (2016). Defective ceramide synthases in mice cause reduced amplitudes in electroretinograms and altered sphingolipid composition in retina and cornea. Eur. J. Neurosci..

[19] Venkataraman K., Riebeling C., Bodennec J., Riezman H., Allegood J.C., Sullards M.C., Merrill A.H., Futerman A.H. (2002). Upstream of growth and differentiation factor 1 (uog1), a mammalian homolog of the yeast longevity assurance gene 1 (LAG1), regulates N-stearoyl-sphinganine (C18-(dihydro)ceramide) synthesis in a fumonisin B1-independent manner in mammalian cells. J. Biol. Chem..

[20] Spassieva S., Seo J.-G., Jiang J.C., Bielawski J., Alvarez-Vasquez F., Jazwinski S.M., Hannun Y.A., Obeid L.M. (2006). Necessary role for the lag1p motif in (dihydro)ceramide synthase activity. J. Biol. Chem..

[21] Senkal C.E., Ponnusamy S., Rossi M.J., Bialewski J., Sinha D., Jiang J.C., Jazwinski S.M., Hannun Y.A., Ogretmen B. (2007). Role of human longevity assurance gene 1 and C18-ceramide in chemotherapy-induced cell death in human head and neck squamous cell carcinomas. Mol. Cancer Ther..

[22] Mullen T.D., Spassieva S., Jenkins R.W., Kitatani K., Bielawski J., Hannun Y.A., Obeid L.M. (2011). Selective knockdown of ceramide synthases reveals complex interregulation of sphingolipid metabolism. J. Lipid Res..

[23] Baran Y., Salas A., Senkal C.E., Gunduz U., Bielawski J., Obeid L.M., Ogretmen B. (2007). Alterations of ceramide/sphingosine-1-phosphate rheostat involved in the regulation of resistance to imatinib-induced apoptosis in K562 human chronic myeloid leukemia cells. J. Biol. Chem..

[24] Wooten-Blanks L.G., Song P., Senkal C.E., Ogretmen B. (2007). Mechanisms of ceramide-mediated repression of the human telomerase reverse transcriptase promoter via deacetylation of Sp3 by histone deacetylase 1. Faseb J..

[25] Mizutani Y., Kihara A., Igarashi Y. (2005). Mammalian Lass6 and its related family members regulate synthesis of specific ceramides. Biochem. J..

[26] Ginkel C., Hartmann D., vom Dorp K., Zlomuzica A., Farwanah H., Eckhardt M., Sandhoff R., Degen J., Rabionet M., Dere E. (2012). Ablation of neuronal ceramide synthase 1 in mice decreases ganglioside levels and expression of myelin-associated glycoprotein in oligodendrocytes. J. Biol. Chem..

[27] Mizutani Y., Kihara A., Chiba H., Tojo H., Igarashi Y. (2008). 2-Hydroxy-ceramide synthesis by ceramide synthase family: Enzymatic basis for the preference of FA chain length. J. Lipid Res..

[28] Jennemann R., Rabionet M., Gorgas K., Epstein S., Dalpke A., Rothermel U., Bayerle A., van der Hoeven F., Imgrund S., Kirsch J. (2012). Loss of ceramide synthase 3 causes lethal skin barrier disruption. Hum. Mol. Genet..

[29] Guillas I., Jiang J.C., Vionnet C., Roubaty C., Uldry D., Chuard R., Wang J., Jazwinski S.M., Conzelmann A. (2003). Human homologues of LAG1 reconstitute Acyl-CoA-dependent ceramide synthesis in yeast. J. Biol. Chem..

[30] Pewzner-Jung Y., Park H., Laviad E.L., Silva L.C., Lahiri S., Stiban J., Erez-Roman R., Brugger B., Sachsenheimer T., Wieland F. (2010). A critical role for ceramide synthase 2 in liver homeostasis: I. Alterations in lipid metabolic pathways. J. Biol. Chem..

[31] Mizutani Y., Kihara A., Igarashi Y. (2006). LASS3 (longevity assurance homologue 3) is a mainly testis-specific (dihydro)ceramide synthase with relatively broad substrate specificity. Biochem. J..

[32] Riebeling C., Allegood J.C., Wang E., Merrill A.H., Futerman A.H. (2003). Two mammalian longevity assurance gene (LAG1) family members, trh1 and trh4, regulate dihydroceramide synthesis using different fatty acyl-CoA donors. J. Biol. Chem..

[33] Ebel P., Imgrund S., Vom Dorp K., Hofmann K., Maier H., Drake H., Degen J., Dormann P., Eckhardt M., Franz T. (2014). Ceramide synthase 4 deficiency in mice causes lipid alterations in sebum and results in alopecia. Biochem. J..

[34] Lahiri S., Futerman A.H. (2005). LASS5 is a bona fide dihydroceramide synthase that selectively utilizes palmitoyl-CoA as acyl donor. J. Biol. Chem..

[35] Gosejacob D., Jager P.S., Vom Dorp K., Frejno M., Carstensen A.C., Kohnke M., Degen J., Dormann P., Hoch M. (2016). Ceramide Synthase 5 Is Essential to Maintain C16:0-Ceramide Pools and Contributes to the Development of Diet-induced Obesity. J. Biol. Chem..

[36] Ebel P., Vom Dorp K., Petrasch-Parwez E., Zlomuzica A., Kinugawa K., Mariani J., Minich D., Ginkel C., Welcker J., Degen J. (2013). Inactivation of ceramide synthase 6 in mice results in an altered sphingolipid metabolism and behavioral abnormalities. J. Biol. Chem..

[37] Goñi F.M., Alonso A. (2002). Sphingomyelinases: Enzymology and membrane activity. FEBS Lett..

[38] Kornhuber J., Rhein C., Muller C.P., Muhle C. (2015). Secretory sphingomyelinase in health and disease. Biol. Chem..

[39] Jenkins R.W., Clarke C.J., Lucas J.T., Shabbir M., Wu B.X., Simbari F., Mueller J., Hannun Y.A., Lazarchick J., Shirai K. (2013). Evaluation of the role of secretory sphingomyelinase and bioactive sphingolipids as biomarkers in hemophagocytic lymphohistiocytosis. Am. J. Hematol..

[40] D’Angelo G., Capasso S., Sticco L., Russo D. (2013). Glycosphingolipids: Synthesis and functions. FEBS J..

[41] Takahashi T., Suzuki T. (2012). Role of sulfatide in normal and pathological cells and tissues. J. Lipid Res..

[42] Lipina C., Hundal H.S. (2015). Ganglioside GM3 as a gatekeeper of obesity-associated insulin resistance: Evidence and mechanisms. FEBS Lett..

[43] Mielke M.M., Haughey N.J. (2012). Could plasma sphingolipids be diagnostic or prognostic biomarkers for Alzheimer’s disease?. Clin. Lipidol..

[44] Mielke M.M., Bandaru V.V.R., Haughey N.J., Rabins P.V., Lyketsos C.G., Carlson M.C. (2010). Serum sphingomyelins and ceramides are early predictors of memory impairment. Neurobiol. Aging.

[45] Savica R., Murray M.E., Persson X.-M., Kantarci K., Parisi J.E., Dickson D.W., Petersen R.C., Ferman T.J., Boeve B.F., Mielke M.M. (2016). Plasma sphingolipid changes with autopsy-confirmed Lewy body or Alzheimer’s pathology. Alzheimers Dement..

[46] Liebisch G., Vizcaino J.A., Kofeler H., Trotzmuller M., Griffiths W.J., Schmitz G., Spener F., Wakelam M.J. (2013). Shorthand notation for lipid structures derived from mass spectrometry. J. Lipid Res..

[47] Han X., Rozen S., Boyle S.H., Hellegers C., Cheng H., Burke J.R., Welsh-Bohmer K.A., Doraiswamy P.M., Kaddurah-Daouk R. (2011). Metabolomics in early Alzheimer’s disease: Identification of altered plasma sphingolipidome using shotgun lipidomics. PLOS ONE.

[48] Kim M., Nevado-Holgado A., Whiley L., Snowden S.G., Soininen H., Kloszewska I., Mecocci P., Tsolaki M., Vellas B., Thambisetty M. (2017). Association between plasma ceramides and phosphatidylcholines and hippocampal brain volume in late onset Alzheimer’s disease. J. Alzheimers Dis..

[49] Mielke M.M., Maetzler W., Haughey N.J., Bandaru V.V., Savica R., Deuschle C., Gasser T., Hauser A.K., Graber-Sultan S., Schleicher E. (2013). Plasma ceramide and glucosylceramide metabolism is altered in sporadic Parkinson’s disease and associated with cognitive impairment: A pilot study. PLOS ONE.

[50] Vidaurre O.G., Haines J.D., Katz Sand I., Adula K.P., Huynh J.L., McGraw C.A., Zhang F., Varghese M., Sotirchos E., Bhargava P. (2014). Cerebrospinal fluid ceramides from patients with multiple sclerosis impair neuronal bioenergetics. Brain.

[51] Pujol-Lereis L.M., Liebisch G., Schick T., Lin Y., Grassmann F., Uchida K., Zipfel P.F., Fauser S., Skerka C., Weber B.H.F. (2018). Evaluation of serum sphingolipids and the influence of genetic risk factors in age-related macular degeneration. PLOS ONE.

[52] Satoi H., Tomimoto H., Ohtani R., Kitano T., Kondo T., Watanabe M., Oka N., Akiguchi I., Furuya S., Hirabayashi Y. (2005). Astroglial expression of ceramide in Alzheimer’s disease brains: A role during neuronal apoptosis. Neuroscience.

[53] Torretta E., Arosio B., Barbacini P., Casati M., Capitanio D., Mancuso R., Mari D., Cesari M., Clerici M., Gelfi C. (2018). Particular CSF sphingolipid patterns identify iNPH and AD patients. Sci. Rep..

[54] Stoessel D., Schulte C., Teixeira Dos Santos M.C., Scheller D., Rebollo-Mesa I., Deuschle C., Walther D., Schauer N., Berg D., Nogueira da Costa A. (2018). Promising metabolite profiles in the plasma and CSF of early clinical Parkinson’s disease. Front. Aging Neurosci..

[55] Pieragostino D., Cicalini I., Lanuti P., Ercolino E., di Ioia M., Zucchelli M., Zappacosta R., Miscia S., Marchisio M., Sacchetta P. (2018). Enhanced release of acid sphingomyelinase-enriched exosomes generates a lipidomics signature in CSF of Multiple Sclerosis patients. Sci. Rep..

[56] Lawton K.A., Cudkowicz M.E., Brown M.V., Alexander D., Caffrey R., Wulff J.E., Bowser R., Lawson R., Jaffa M., Milburn M.V. (2012). Biochemical alterations associated with ALS. Amyotroph. Lateral Scler..

[57] Hannich J.T., Umebayashi K., Riezman H. (2011). Distribution and functions of sterols and sphingolipids. Cold Spring Harb. Perspect. Biol..

[58] Boillée S., Vande Velde C., Cleveland D.W. (2006). ALS: A disease of motor neurons and their nonneuronal neighbors. Neuron.

[59] Zhang J., Zhang X., Wang L., Yang C. (2017). High performance liquid chromatography-mass spectrometry (LC-MS) based quantitative lipidomics study of ganglioside-NANA-3 plasma to establish its association with Parkinson’s disease patients. Med. Sci. Monit..

[60] Chiasserini D., Paciotti S., Eusebi P., Persichetti E., Tasegian A., Kurzawa-Akanbi M., Chinnery P.F., Morris C.M., Calabresi P., Parnetti L. (2015). Selective loss of glucocerebrosidase activity in sporadic Parkinson’s disease and dementia with Lewy bodies. Mol. Neurodegener..

[61] Mielke M.M., Bandaru V.V., Haughey N.J., Xia J., Fried L.P., Yasar S., Albert M., Varma V., Harris G., Schneider E.B. (2012). Serum ceramides increase the risk of Alzheimer disease: The Women’s Health and Aging Study II. Neurology.

[62] Mielke M.M., Haughey N.J., Han D., An Y., Bandaru V.V.R., Lyketsos C.G., Ferrucci L., Resnick S.M. (2017). The association between plasma ceramides and sphingomyelins and risk of Alzheimer’s disease differs by sex and APOE in the Baltimore Longitudinal Study of Aging. J. Alzheimers Dis..

[63] Liang Q., Liu H., Zhang T., Jiang Y., Xing H., Zhang A.-H. (2016). Discovery of serum metabolites for diagnosis of progression of mild cognitive impairment to Alzheimer’s disease using an optimized metabolomics method. RSC Advances.

[64] Spiegel S., Milstien S. (2011). The outs and the ins of sphingosine-1-phosphate in immunity. Nat. Rev. Immunol..

[65] Han X., Holtzman D.M., McKeel D., Kelley J., Morris J.C. (2002). Substantial sulfatide deficiency and ceramide elevation in very early Alzheimer’s disease: Potential role in disease pathogenesis. J. Neurochem..

[66] Chan R.B., Oliveira T.G., Cortes E.P., Honig L.S., Duff K.E., Small S.A., Wenk M.R., Shui G., Di Paolo G. (2012). Comparative lipidomic analysis of mouse and human brain with Alzheimer disease. J. Biol. Chem..

[67] Cutler R.G., Kelly J., Storie K., Pedersen W.A., Tammara A., Hatanpaa K., Troncoso J.C., Mattson M.P. (2004). Involvement of oxidative stress-induced abnormalities in ceramide and cholesterol metabolism in brain aging and Alzheimer’s disease. Proc. Natl. Acad. Sci. USA..

[68] Panchal M., Gaudin M., Lazar A.N., Salvati E., Rivals I., Ayciriex S., Dauphinot L., Dargere D., Auzeil N., Masserini M. (2014). Ceramides and sphingomyelinases in senile plaques. Neurobiol. Dis..

[69] Abbott S.K., Li H., Muñoz S.S., Knoch B., Batterham M., Murphy K.E., Halliday G.M., Garner B. (2014). Altered ceramide acyl chain length and ceramide synthase gene expression in Parkinson’s disease. Mov. Disord..

[70] Ben-David O., Futerman A.H. (2010). The role of the ceramide acyl chain length in neurodegeneration: Involvement of ceramide synthases. Neuromol. Med..

[71] Imgrund S., Hartmann D., Farwanah H., Eckhardt M., Sandhoff R., Degen J., Gieselmann V., Sandhoff K., Willecke K. (2009). Adult ceramide synthase 2 (CERS2)-deficient mice exhibit myelin sheath defects, cerebellar degeneration, and hepatocarcinomas. J. Biol. Chem..

[72] Gerstl B., Eng L.F., Tavaststjerna M., Smith J.K., Kruse S.L. (1970). Lipids and proteins in multiple sclerosis white matter. J. Neurochem..

[73] O’Gorman C., Lucas R., Taylor B. (2012). Environmental risk factors for multiple sclerosis: A review with a focus on molecular mechanisms. Int. J. Mol. Sci..

[74] Astarita G., Jung K.-M., Vasilevko V., DiPatrizio N.V., Martin S.K., Cribbs D.H., Head E., Cotman C.W., Piomelli D. (2011). Elevated stearoyl-CoA Desaturase in Brains of Patients with Alzheimer’s disease. PLOS ONE.

[75] Wood P.L. (2012). Lipidomics of Alzheimer’s disease: Current status. Alzheimers Res. Ther..

[76] Seumois G., Fillet M., Gillet L., Faccinetto C., Desmet C., Francois C., Dewals B., Oury C., Vanderplasschen A., Lekeux P. (2007). *De novo* C16- and C24-ceramide generation contributes to spontaneous neutrophil apoptosis. J. Leukoc. Biol..

[77] Osawa Y., Uchinami H., Bielawski J., Schwabe R.F., Hannun Y.A., Brenner D.A. (2005). Roles for C16-ceramide and sphingosine-1-phosphate in regulating hepatocyte apoptosis in response to tumor necrosis factor-alpha. J. Biol. Chem..

[78] Chen H., Tran J.-T.A., Brush R.S., Saadi A., Rahman A.K., Yu M., Yasumura D., Matthes M.T., Ahern K., Yang H. (2012). Ceramide signaling in retinal degeneration. Adv. Exp. Med. Biol..

[79] Rudd A.K., Devaraj N.K. (2018). Traceless synthesis of ceramides in living cells reveals saturation-dependent apoptotic effects. Proc. Natl. Acad. Sci. USA.

[80] Grosch S., Schiffmann S., Geisslinger G. (2012). Chain length-specific properties of ceramides. Prog. Lipid Res..

[81] Mesicek J., Lee H., Feldman T., Jiang X., Skobeleva A., Berdyshev E.V., Haimovitz-Friedman A., Fuks Z., Kolesnick R. (2010). Ceramide synthases 2, 5, and 6 confer distinct roles in radiation-induced apoptosis in HeLa cells. Cell. Signal..

[82] Ten Grotenhuis E., Demel R.A., Ponec M., Boer D.R., van Miltenburg J.C., Bouwstra J.A. (1996). Phase behavior of stratum corneum lipids in mixed Langmuir-Blodgett monolayers. Biophys. J..

[83] Siskind L.J., Mullen T.D., Romero Rosales K., Clarke C.J., Hernandez-Corbacho M.J., Edinger A.L., Obeid L.M. (2010). The BCL-2 protein BAK is required for long-chain ceramide generation during apoptosis. J. Biol. Chem..

[84] Reichel M., Rhein C., Hofmann L.M., Monti J., Japtok L., Langgartner D., Füchsl A.M., Kleuser B., Gulbins E., Hellerbrand C. (2018). Chronic psychosocial stress in mice is associated with increased acid sphingomyelinase activity in liver and serum and with hepatic C16:0-ceramide accumulation. Front. Psychiatry.

[85] Jenkins R.W., Canals D., Idkowiak-Baldys J., Simbari F., Roddy P., Perry D.M., Kitatani K., Luberto C., Hannun Y.A. (2010). Regulated secretion of acid sphingomyelinase: Implications for selectivity of ceramide formation. J. Biol. Chem..

[86] Sassa T., Suto S., Okayasu Y., Kihara A. (2012). A shift in sphingolipid composition from C24 to C16 increases susceptibility to apoptosis in HeLa cells. Biochim. Biophys. Acta.

[87] Spassieva S.D., Mullen T.D., Townsend D.M., Obeid L.M. (2009). Disruption of ceramide synthesis by CerS2 down-regulation leads to autophagy and the unfolded protein response. Biochem. J..

[88] Spassieva S.D., Ji X., Liu Y., Gable K., Bielawski J., Dunn T.M., Bieberich E., Zhao L. (2016). Ectopic expression of ceramide synthase 2 in neurons suppresses neurodegeneration induced by ceramide synthase 1 deficiency. Proc. Natl. Acad. Sci. USA.

[89] Petersen M.C., Shulman G.I. (2018). Mechanisms of insulin action and insulin resistance. Physiol. Rev..

[90] Turpin S.M., Nicholls H.T., Willmes D.M., Mourier A., Brodesser S., Wunderlich C.M., Mauer J., Xu E., Hammerschmidt P., Brönneke H.S. (2014). Obesity-Induced CerS6-Dependent C16:0 Ceramide Production Promotes Weight Gain and Glucose Intolerance. Cell Metab..

[91] Haus J.M., Kashyap S.R., Kasumov T., Zhang R., Kelly K.R., Defronzo R.A., Kirwan J.P. (2009). Plasma ceramides are elevated in obese subjects with type 2 diabetes and correlate with the severity of insulin resistance. Diabetes.

[92] Straczkowski M., Kowalska I., Nikolajuk A., Dzienis-Straczkowska S., Kinalska I., Baranowski M., Zendzian-Piotrowska M., Brzezinska Z., Gorski J. (2004). Relationship between insulin sensitivity and sphingomyelin signaling pathway in human skeletal muscle. Diabetes.

[93] Coen P.M., Dubé J.J., Amati F., Stefanovic-Racic M., Ferrell R.E., Toledo F.G.S., Goodpaster B.H. (2010). Insulin resistance is associated with higher intramyocellular triglycerides in type I but not type II myocytes concomitant with higher ceramide content. Diabetes.

[94] Kirwan J.P. (2013). Plasma ceramides target skeletal muscle in type 2 diabetes. Diabetes.

[95] Norheim F., Bjellaas T., Hui S.T., Chella Krishnan K., Lee J., Gupta S., Pan C., Hasin-Brumshtein Y., Parks B.W., Li D.Y. (2018). Genetic, dietary, and sex-specific regulation of hepatic ceramides and the relationship between hepatic ceramides and IR. J. Lipid Res..

[96] Fehm H.L., Kern W., Peters A. (2006). The selfish brain: Competition for energy resources. Prog. Brain Res..

[97] Dineley K.T., Jahrling J.B., Denner L. (2014). Insulin resistance in Alzheimer’s disease. Neurobiol. Dis..

[98] Craft S. (2009). The role of metabolic disorders in Alzheimer disease and vascular dementia: Two roads converged. Arch. Neurol..

[99] Chiu C.J., Taylor A. (2011). Dietary hyperglycemia, glycemic index and metabolic retinal diseases. Prog. Retin. Eye. Res..

[100] Chen X., Rong S.S., Xu Q., Tang F.Y., Liu Y., Gu H., Tam P.O., Chen L.J., Brelen M.E., Pang C.P. (2014). Diabetes mellitus and risk of age-related macular degeneration: A systematic review and meta-analysis. PLOS ONE.

[101] Tarchick M.J., Cutler A.H., Trobenter T.D., Kozlowski M.R., Makowski E.R., Holoman N., Shao J., Shen B., Anand-Apte B., Samuels I.S. (2018). Endogenous insulin signaling in the RPE contributes to the maintenance of rod photoreceptor function in diabetes. Exp. Eye Res..

[102] Leontieva O.V., Demidenko Z.N., Blagosklonny M.V. (2014). Rapamycin reverses insulin resistance (IR) in high-glucose medium without causing IR in normoglycemic medium. Cell Death Dis..

[103] Sánchez-Chávez G., Peña-Rangel M.T., Riesgo-Escovar J.R., Martínez-Martínez A., Salceda R. (2012). Insulin stimulated-glucose transporter Glut 4 is expressed in the retina. PLOS ONE.

[104] Mantych G.J., Hageman G.S., Devaskar S.U. (1993). Characterization of glucose transporter isoforms in the adult and developing human eye. Endocrinology.

[105] Lötsch J., Thrun M., Lerch F., Brunkhorst R., Schiffmann S., Thomas D., Tegder I., Geisslinger G., Ultsch A. (2017). Machine-learned data structures of lipid marker serum concentrations in multiple sclerosis patients differ from those in healthy subjects. Int. J. Mol. Sci..

[106] Gurke R., Etyemez S., Prvulovic D., Thomas D., Fleck S.C., Reif A., Geisslinger G., Lötsch J. (2019). A data science-based analysis points at distinct patterns of lipid mediator plasma concentrations in patients with dementia. Front. Psychiatry.

[107] Lötsch J., Schiffmann S., Schmitz K., Brunkhorst R., Lerch F., Ferreiros N., Wicker S., Tegeder I., Geisslinger G., Ultsch A. (2018). Machine-learning based lipid mediator serum concentration patterns allow identification of multiple sclerosis patients with high accuracy. Sci. Rep..

[108] Rajesh M., Kolmakova A., Chatterjee S. (2005). Novel role of lactosylceramide in vascular endothelial growth factor-mediated angiogenesis in human endothelial cells. Circ. Res..

[109] Birklé S., Desselle A., Chaumette T., Gaugler M.-H., Cochonneau D., Fleurence J., Dubois N., Hulin P., Aubry J., Paris F. (2013). Inhibition of tumor angiogenesis by globotriaosylceramide immunotargeting. Oncoimmunology.

[110] Bandaru V.V.R., Troncoso J., Wheeler D., Pletnikova O., Wang J., Conant K., Haughey N.J. (2009). ApoE4 disrupts sterol and sphingolipid metabolism in Alzheimer’s but not normal brain. Neurobiol. Aging.

